# Autism and Piperacillin-tazobactam-induced Hypersensitivity Vasculitis: A Silent Malady

**DOI:** 10.7759/cureus.4574

**Published:** 2019-04-30

**Authors:** Sindhura Kolli, Srilaxmi Gujjula

**Affiliations:** 1 Internal Medicine, The Brooklyn Hospital Center, Affiliate of the Mount Sinai Hospital, Brooklyn, USA

**Keywords:** zosyn, piperacillin tazobactam, autism spectrum disorder, autism

## Abstract

Autism spectrum disorder (ASD) is a disorder affecting communication, with behaviors such as hyporesponsiveness to stimuli. When coupled with a lower threshold for allergic reactions, it can lead to a delayed identification of life-threatening anaphylaxis. It can also delay treatment for lesser complications such as with our patient, who developed a pruritic, erythematous rash as direct causation from piperacillin-tazobactam. This case addresses the delays in clinical care when approaching the administration of new medication in patients affected by autism.

## Introduction

Autism spectrum disorder (ASD) is a disorder requiring a multidisciplinary approach due to the presence of persistent deficits in communication coupled with a higher prevalence of comorbidities such as infections and allergic reactions. In our case, we demonstrate the clinical spectrum of hypersensitivity vasculitis in response to piperacillin-tazobactam and explain how to approach it in patients affected by autism.

## Case presentation

A 48-year-old Caucasian male, with a past medical history of autism with speech impairments and epilepsy, presented to the emergency room with a fever of 100.6 degrees Fahrenheit (F) and an unintentional weight loss of 30 pounds (lbs). A septic workup was initiated, which included a chest X-ray displaying a new right lower lung opacity. Blood and urine cultures were negative and the patient was unable to give an adequate respiratory culture sample. Given his history of recently being hospitalized prior to admission, hospital-acquired pneumonia became the working diagnosis. Piperacillin-tazobactam (Zosyn®) was initiated before morning rounds.

**Figure 1:**
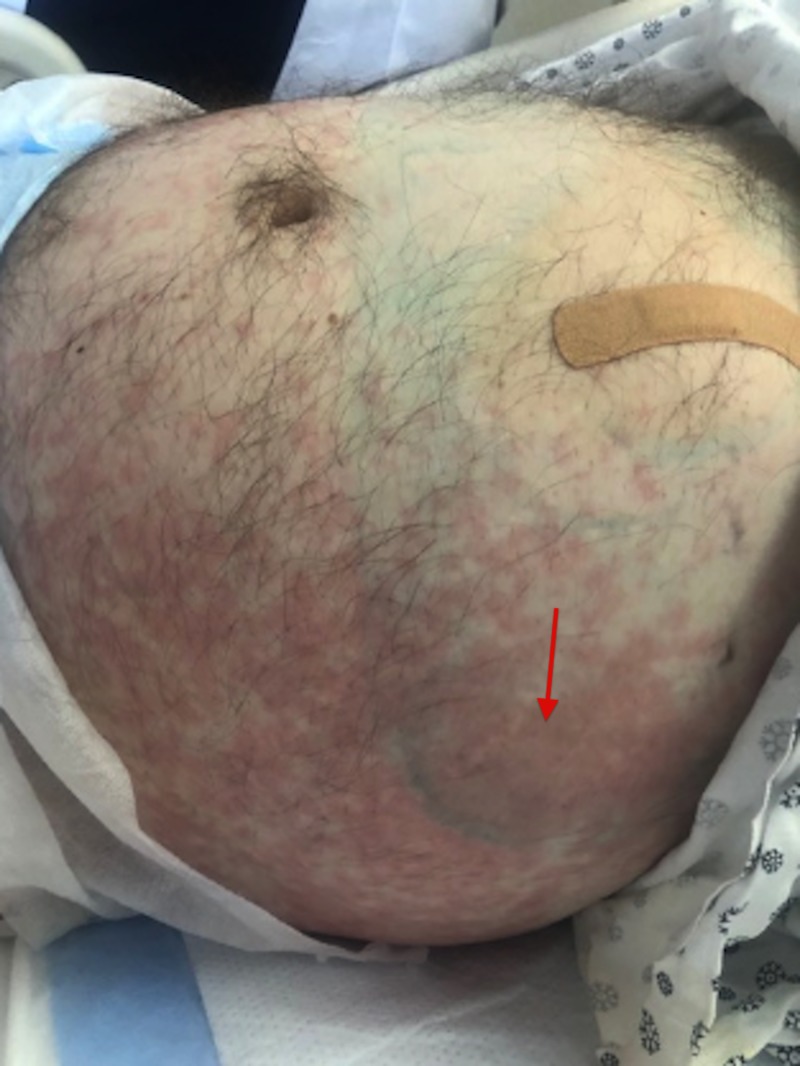
Zosyn-induced hypersensitivity vasculitis along the abdomen and flanks extending to the groin

**Figure 2:**
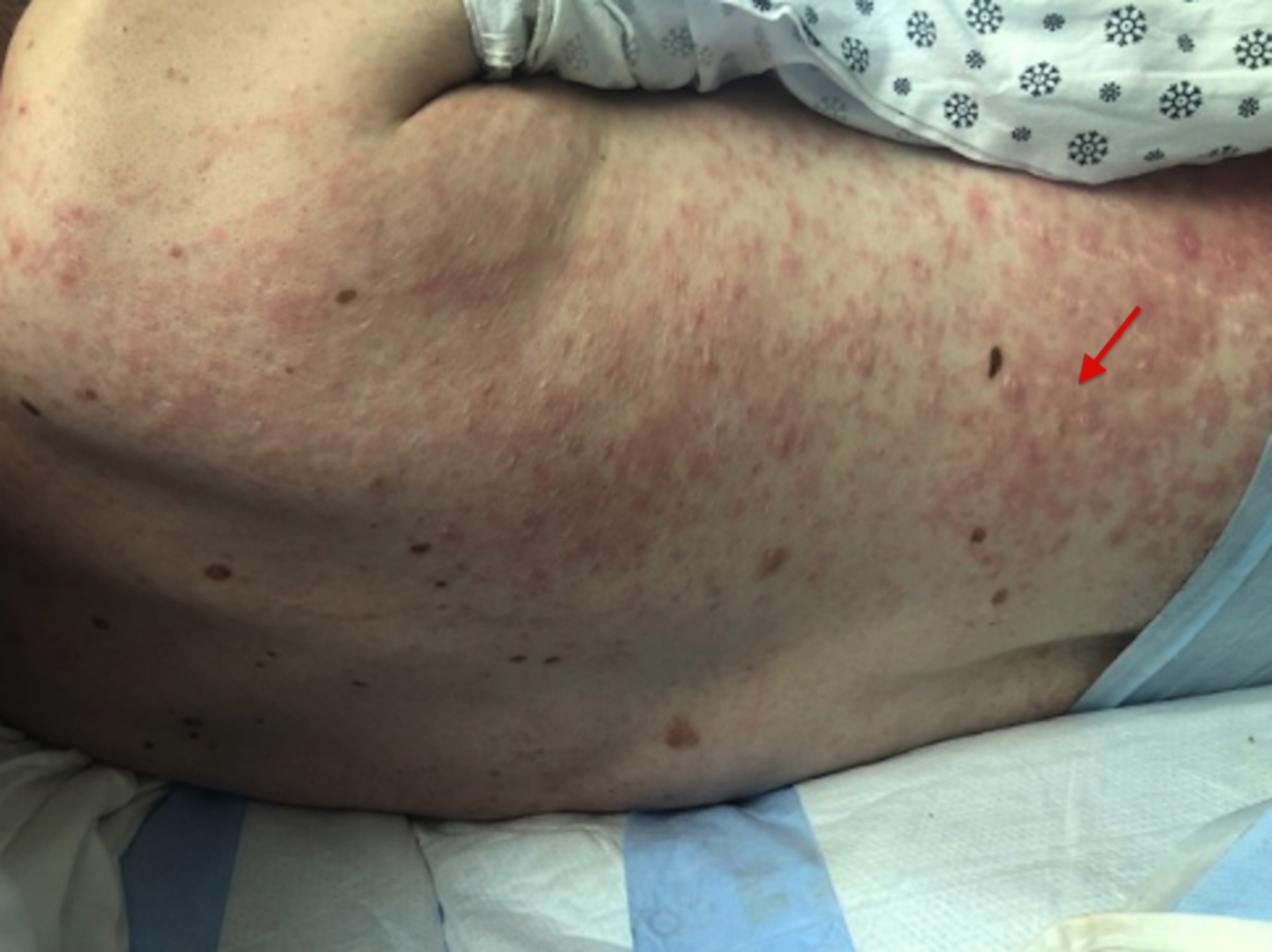
Zosyn-induced maculopapular rash with excoriations

Approximately 24 hours later, the patient was found to have a diffuse maculopapular erythematous rash along the flanks and abdomen, extending to the groin and lower medial thighs as well as his back (Figures 1-2). The patient had mild pruritus, but due to his developmental delays, had difficulty communicating symptoms and brought no attention to it. The patient was afebrile, with absent eosinophilia and a normal platelet count. Zosyn was immediately discontinued and replaced with aztreonam and metronidazole. Methylprednisolone and diphenhydramine, an H1-antagonist, were given.

Daily improvement of the rash and pruritus was observed over the course of the week. Workup for fevers and unexplained weight loss revealed moderately differentiated adenocarcinoma with extensive necrosis, likely cholangiocarcinoma. The patient was discharged with follow-up with oncology.

## Discussion

Autism is a disorder characterized by difficulty in communication and repetitive behavioral patterns. Sensory patterns that co-exist in patients with autism consist of hyporesponsiveness, which are mitigated or delayed responses to stimuli, and sensory seeking, which is a fascination with unusual stimuli. Both sensory patterns have a link with poor development of communication skills in people with autism [1]. People with autism spectrum disease (ASD) have a higher prevalence of allergies and allergic responses, which in turn are involved in ASD neuropathogenesis and routinely underdiagnosed [2]. This association is due to the dysfunction of B cells, T-helper (Th) 1 and Th2 lymphocytes, natural killer (NK) cells, increased cytokines, and auto-antibodies, resulting in a pro-inflammatory state and hyperactivity upon contact with an allergen [3-4]. In this case, the allergen was piperacillin-tazobactam, otherwise known as Zosyn.

Zosyn is a broad-spectrum antibiotic used for intra-abdominal infection, urinary tract infections, skin, and soft tissue infections, or, like in our patient, lower respiratory infections. In the North American phase, in three clinical monotherapy trials with Zosyn, 90% of adverse events (AEs) were found to be transient in nature. They ranged from headaches (7.7%), insomnia (6.6%), myalgias (<1%), flushing (<1%), hypotension (<1%), injection site reaction (<1%), hypoglycemia (<1%), and epistaxis (<1%). These rare and transient side effects have led to worldwide acceptance and frequent use of the drug. Any discontinuation of the medication stems from its more severe AEs involving the gastrointestinal tract and the skin, such as when diarrhea (11.3%), dyspepsia (3.3%), vomiting (3.3%), and/or anaphylaxis (<1%) occur. It is also discontinued when hypersensitivity vasculitis (HV), seen as rashes of the maculopapular, bullous or urticarial type (4.2%), pruritus (3.1%), or purpura (<1%) appears [5], like in our patient.

Hypersensitivity vasculitis is the inflammation and damage of blood vessels in the skin in reaction to a drug. The American Association of Rheumatology has definitive criteria for HV as i) age at disease onset >16 years, ii) medication taken at the onset of symptoms, iii) non-blanchable palpable purpura not associated with thrombocytopenia, iv) presence of maculopapular rash demonstrated by flat and raised lesions of various sizes, v) biopsy demonstrating neutrophils in the vessel wall, and vi) biopsy demonstrating eosinophils. When all criteria are fulfilled, this denotes sensitivity of 78.5% and specificity of 78.7% [6] of being HV. Our patient refused a biopsy and fulfilled four out of the six criteria when Zosyn was given. However, because of the sensory patterns and communication deficits associated with ASD, our patient was unable to communicate the symptoms of HV, delaying immediate discontinuation.

## Conclusions

When initiating a new medication with potential anaphylactic or hypersensitivity reactions in a patient with ASD, it is advisable to stay with the patient to perceive any immediate reactions and increase the frequency of checks within the first 24 hours to catch AEs at an earlier stage for ceasing offending agents and starting treatment earlier. Patients with ASD are less likely to communicate the symptoms caused by a new drug agent until frank signs or complications are perceived by the physician and thus would need to be monitored closely. Increasing the frequency of rounding on patients with ASD started on new medications would likely prevent AEs and reduce the sequelae of worsening complications, increase compliance within a patient with ASD, and decrease the aggravation in an ASD patient caused by unpleasant symptoms compounded by the inability to communicate them.
